# Bridging the gap—Immune cells that can repair nerves

**DOI:** 10.1038/s41423-021-00642-7

**Published:** 2021-02-12

**Authors:** Helen M. McGettrick

**Affiliations:** Institute of Inflammation and Aging, University of Birmingham, Birmingham, UK

**Keywords:** Neuroimmunology, Mechanisms of disease

Immunity is critical for survival in a world plagued by pathogens. Like that of most military operations, the strategy of the immune system is based on acceptable losses: determining the maximal amount of damage to uninfected tissues that is necessary to defeat or entrap any invading pathogen. This characteristic has devastating consequences for patients with immune-mediated diseases, including many neurological conditions. However, nature often creates a system of “checks and balances”, and the immune system is no exception, having developed a repair strategy to mitigate unwanted tissue damage resulting from leukocyte responses to microbes. It is unclear whether such strategies are also used at sites of sterile injury. Sas and colleagues recently described a novel neutrophil subpopulation that is capable of promoting nerve repair following acute injury.^1^

Immunosuppressive leukocytes were first described in 1977, in which Kung demonstrated that myeloid cells expressing arginase 1 (Arg1) were capable of suppressing cytotoxic responses.^2^ Over the last 40+ years, a growing body of evidence has attributed immunosuppressive or regenerative properties to subpopulations of myeloid cells that express Arg1 and the mannose receptor (CD206), coining the terms alternatively activated cells, unconventional cells, M2 macrophages and N2 neutrophils. These cellular functions can be replicated in vitro by polarizing myeloid cells with cytokines or growth factors: monocytes stimulated with IL-4 or IL-13^3^ and neutrophils stimulated with TGFβ or GM-CSF.^4^ Furthermore, the presence of these cells has been described at sites of wound repair, such as the skin^5^ and spinal cord,^6^ and these cells are thought to be an important source of resolution and reparative factors, such as IL-10 and TGFβ.

The infiltration of proinflammatory leukocytes into the central nervous system (CNS) is considered detrimental in the context of multiple sclerosis (MS^7^) and stroke.^8^ But this represents only half of the story: CNS-resident macrophages (microglia) with an M2-like phenotype (Arg1^+^) are present in MS patients entering clinical remission.^9^ Indeed, the switch to the M2 microglial phenotype has been reported to coincide with the differentiation of murine progenitor cells into myelin sheath-forming oligodendrocytes to repair regions of toxin-induced demyelination in the brain.^10^ Other studies have suggested that it is the early infiltration of Ly6G^+^ neutrophils, rather than monocytes, who secrete regenerative factors (e.g., oncomodulin^10^) and are therefore the principal regulators of optic nerve regeneration^10^ and spinal cord repair [e.g.,^6^]. Despite these early studies, gaps in our knowledge remain, such as which signals promote neutrophils to switch from tissue-damaging to tissue-repairing functions.

To address this knowledge gap, Sas et al. used a CNS injury and regeneration model in which the optic nerve was crushed, resulting in neuronal death that was rescued by intraocular zymosan, which triggers myeloid cell repair processes.^11,12^ Using this optic nerve crush zymosan model, Segal and colleagues revealed that neutrophils were the predominant cell type in the vitreous fluid during the critical timeframe for stimulating retinal ganglion cell repair and axon regeneration. Immunofluorescence imaging was used to count the number of regenerating axons in optic nerve sections and the distance of these axons from the crush site. Importantly, the researchers observed this repair response in T and B cell-deficient (*Rag1*^−/−^) mice, indicating that myeloid cells were responsible for driving this response. To confirm this finding, Segal and colleagues attempted to block the entry of mature neutrophils into the injured eye by systemically injecting a functional blocking antibody against CXCR2 immediately after inducing optic nerve crush and administering zymosan. Rather than observing the loss of axon regeneration due to a reduce neutrophil infiltrate in the eye, anti-CXCR2 treatment enhanced the number of axons within and extending away from the crush site, and also delayed the infiltration of myeloid cells expressing the neutrophil marker CD11b - raising the question: what were these cells?

Using flow cytometric analysis, Sas et al. revealed that anti-CXCR2 treatment triggered the expansion of a population of arginase 1-expressing neutrophils in the blood and vitreous fluid that shared characteristics associated with immature neutrophils (CD14^+^Ly6G^low^CD101^low-neg^ with ring-shaped nuclei). Single-cell RNA-seq analysis of Ly6G^+^ cells confirmed these findings and revealed an abundance of *Arg1*, *Mrc1*, and *Il4ra* transcripts, which are associated with N2 neutrophils, that were enriched in the immature neutrophil cluster. Furthermore, the group demonstrated that intraocular, but not blood, Ly6G^+^ cells were capable of stimulating neurite outgrowth when cocultured with primary retinal ganglion cells in vitro.

The low yield of purified Ly6G^low^ cells from vitreous fluid prohibited their use in adoptive transfer experiments. As an alternative, Segal and colleagues adoptively transferred peritoneal neutrophils obtained 3 days after zymosan-induced peritonitis (3d neutrophils). First, the group validated that 3d neutrophils shared similar traits to intraocular neutrophils harvested from the optic nerve crush zymosan model following anti-CXCR2 treatment, including relatively low surface expression of Ly6G and CD101 and high expression of *Arg1*, *Mrc1*, and *Il4ra* transcripts. Crucially, these cells stimulated an increase in the number of axons observed within and extending away from the crush site when adoptively transferred directly into the eyes of mice with optic nerve crush. These results were consistent the effects that Sas et al. had previously observed when zymosan and anti-CXCR2 treatment were administered at the time of optic nerve crush injury. Validating adoptive transfer studies in transgenic mice confirmed that this response was specific to these cells and not due to free zymosan particles or the polarization of monocytes within the eye to an M2 phenotype.

Two questions remained unanswered: What are the regenerative factors/signaling pathways involved? Is this response limited to the eye microenvironment or shared across neuronal tissues? To address these questions, Segal and colleagues first cultured retinal ganglion cells or dorsal ganglia cells with 3d neutrophils or their conditioned media and observed axonal outgrowth of neurons in all contexts. Moreover, the researchers used a traumatic spinal cord injury model to show that 3d neutrophils were able to stimulate the regrowth of severed axons beyond the injury site, highlighting the neuroprotective and regenerative functions of 3d neutrophils in both the optic nerve and spinal cord. Subsequent multiplex, RT-qPCR and ELISA analyses revealed high levels of nerve growth factor (NGF) and insulin-like growth factor-1 (IGF-1) in the conditioned media of 3d neutrophils, and also in vitreous fluid following optic nerve crush plus zymosan, which were further increased in response to anti-CXCR2 treatment. A series of loss-of-function and gain-of-function experiments in vitro and in vivo confirmed that NGF and IGF-1 were the pro-regenerative agents responsible, in part, for axon regeneration following optic nerve crush injury.

Finally, the group assessed the capacity of human immature neutrophils to initiate neuronal repair. The researchers took advantage of the fact that upon short-term resting culture with DMSO (dimethylsulfoxide) the human promyelocytic leukemia cell line (HL-60) exhibits a cell surface antigen profile indicative of immature neutrophils, expressing high levels of *Arg1* and *NGF* transcripts and secreting NGF. The group showed that these HL-60 cells were able stimulate axon regeneration when adoptively transferred into the eyes of *Rag1*-deficient mice with optic nerve crush injury or when cocultured with primary retinal ganglion cells in vitro. Neutralizing HL-60 cell-secreted NGF partly reduced neurite outgrowth in vitro, indicating that the reparative potential of immature human granulocytes is mediated in part by a growth factor-dependent mechanism.

**Fig. 1:**
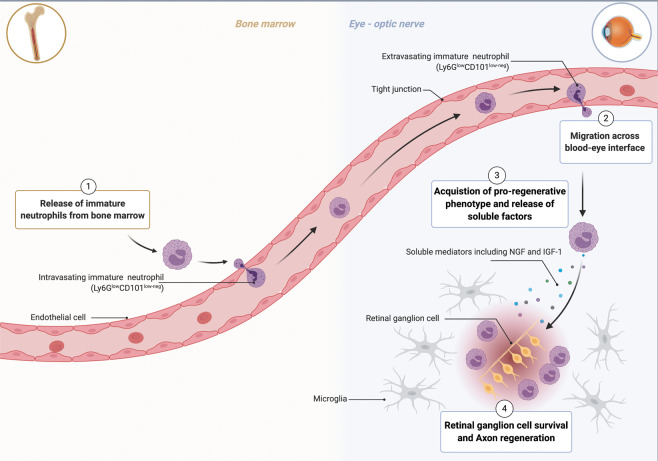
Regenerative neutrophils stimulate optic nerve repair. Optic nerve crush injury results in neuronal death that is rescued by intraocular zymosan administration. This combination of injury and inflammation triggers the premature release of immature neutrophils from the bone marrow (1). These neutrophils (Ly6G^low^CD101^low-neg^) are recruited to the eye in a CXCR2-independent manner (2) where they receive signals that stimulate the acquisition of a regenerative phenotype (3). These regenerative neutrophils secrete repair factors, including nerve growth factors (NGF) and insulin-like growth factor-1 (IGF-1), that stimulate retinal ganglion cell survival and axon regeneration (4). Created with BioRender.com

The take-home message from the study is the existence of a novel myeloid/granulocyte population with neuroprotective and axon regenerative properties in the context of acute optical nerve (Fig. 1) and spinal cord injury.^1^

Some questions remain unanswered. Clearly, the study examined only one human cell line, HL-60, while immunosuppressive Arg1^+^ primary human neutrophils have been isolated and described in several disease states^13,14^ and in pregnancy.^15^ It will be important to ascertain the regenerative properties of these cells before screening for their presence in patients with CNS injury. Studies are needed to determine the other soluble mediators acting alongside NGF and IGF-1 to mediate pro-axon regeneration and the significance, if any, of the response of glial cells to these agents or to the regenerative neutrophils in this neuroprotective process. Notably, blood Ly6G^low^ cells do not exhibit these reparative properties. Therefore, which signals (e.g., chemokines) do these cells encounter as they migrate into the injured site that allow for the acquisition of this function? Such future work will have huge implications in the development of neuroprotective, regenerative immunotherapies, which could involve the generation of autologous regenerative neutrophil-based therapies and/or biological agents to promote the selective migration of endogenous regenerative neutrophils or the acquisition of this phenotype in the injured site.
